# Bromido(2,4,6-trimethyl­phen­yl)mercury(II)

**DOI:** 10.1107/S1600536812010392

**Published:** 2012-03-14

**Authors:** Frank Meyer-Wegner, Hans-Wolfram Lerner, Tanja Sinke, Michael Bolte

**Affiliations:** a Institut für Anorganische und Analytische Chemie, Goethe-Universität Frankfurt, Max-von-Laue-Strasse 7, 60438 Frankfurt am Main, Germany

## Abstract

Mol­ecules of the title compound, [HgBr(C_9_H_11_)], are located on a crystallographic twofold rotation axis. Due to the mol­ecular symmetry, the Hg^II^ atom is linearly coordinated by the *ipso*-C of the mesityl group and the Br atom. In the crystal, mol­ecules lie in planes parallel to (001).

## Related literature

For dimesityl-mercury, see: Hayashi *et al.* (2011 ▶). For the synthesis of Hg[Mes]_2_, see: Hübner *et al.* (2010 ▶).
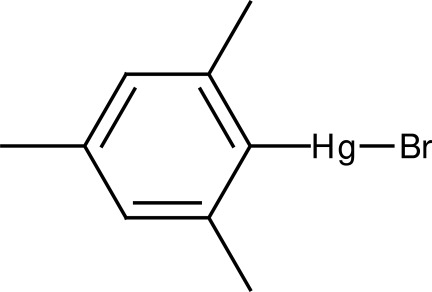


## Experimental

### 

#### Crystal data

[HgBr(C_9_H_11_)]
*M* *_r_* = 399.68
Monoclinic, 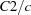

*a* = 10.0459 (8) Å
*b* = 15.3072 (13) Å
*c* = 8.1517 (7) Å
β = 126.912 (5)°
*V* = 1002.27 (14) Å^3^
*Z* = 4
Mo *K*α radiation
μ = 19.28 mm^−1^
*T* = 173 K
0.21 × 0.10 × 0.03 mm

#### Data collection

Stoe IPDS II two-circle diffractometer
Absorption correction: multi-scan (*MULABS*; Spek, 2009 ▶; Blessing, 1995 ▶) *T*_min_ = 0.107, *T*_max_ = 0.595
6756 measured reflections
942 independent reflections
892 reflections with *I* > 2σ(*I*)
*R*_int_ = 0.103

#### Refinement

*R*[*F* ^2^ > 2σ(*F* ^2^)] = 0.025
*wR*(*F* ^2^) = 0.057
*S* = 1.05
942 reflections
55 parameters
H-atom parameters constrained
Δρ_max_ = 1.19 e Å^−3^
Δρ_min_ = −0.71 e Å^−3^

### 

Data collection: *X-AREA* (Stoe & Cie, 2001 ▶); cell refinement: *X-AREA*; data reduction: *X-RED32*; program(s) used to solve structure: *SHELXS97* (Sheldrick, 2008 ▶); program(s) used to refine structure: *SHELXL97* (Sheldrick, 2008 ▶); molecular graphics: *XP* (Sheldrick, 2008 ▶); software used to prepare material for publication: *SHELXL97*.

## Supplementary Material

Crystal structure: contains datablock(s) I, global. DOI: 10.1107/S1600536812010392/tk5065sup1.cif

Structure factors: contains datablock(s) I. DOI: 10.1107/S1600536812010392/tk5065Isup2.hkl

Additional supplementary materials:  crystallographic information; 3D view; checkCIF report

## Figures and Tables

**Fig. 1.**
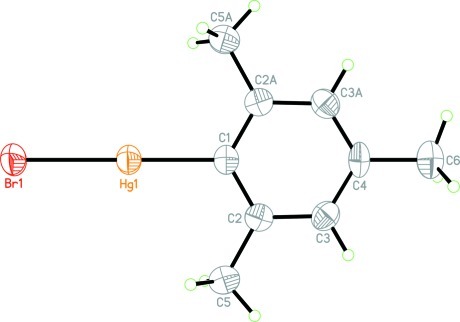
A perspective view of the title compound, showing the atom-numbering scheme. Displacement ellipsoids are drawn at the 50% probability level and H atoms are shown as small spheres of arbitrary radii. Only one set of the H atoms of the disordered methyl group is shown. Unlabelled atoms are related by the symmetry operation 1-x, y, 3/2-z.

**Fig. 2.**
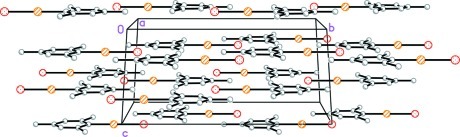
Packing diagram of the title compound viewed onto the bc plane. H atoms omitted for clarity.

## supplementary crystallographic information

### Comment

Very recently we have shown that Hg[Mes]_2_ could be synthesized when HgCl_2_ was treated with two equivalents of Li[Mes] (Hübner *et al.*, 2010) in thf at ambient temperature (Hayashi *et al.*, 2011). In addition, we have investigated the reaction of Hg[Mes]_2_ with BBr_3_ (Hayashi *et al.*, 2011). In this paper we report the structure of the analogous Grignard compound Hg[Mes]Br which was obtained from the 1: 1 reaction of Hg[Mes]_2_ with BBr_3_.

Molecules of the title compound are located on a crystallographic twofold rotation axis with half a molecule in the asymmetric unit. Due to the molecular symmetry, the Hg centre is linearly coordinated by the *ipso*-C of the mesityl group and the Br atom. The Hg—C bond [2.053 (7) Å] is slightly shorter than that in dimesityl mercury [2.080 (6) Å] (Hübner *et al.*, 2010).

In the crystal, the molecules lie in planes parallel to (0 0 1). In a plane, the molecules are oriented parallel to each other with the Hg—Br vectors pointing in the same direction. The shortest intermolecular Hg···Br contact is 4.1270 (4) Å and the shortest intermolecular Hg···Hg contact is 5.1078 (4) Å (symmetry operator for equivalent atoms: 1 - *x*, 1 - *y*, 1 - *z*).

### Experimental

In a round bottom flask Hg[Mes]_2_ (0.21 g, 0.48 mmol) in 40 ml benzene was treated with one equivalent of BBr_3_ (0.046 ml, 120 mg, 0.48 mmol) at ambient temperature. Single crystals of the title compound Hg[Mes]Br were obtained from a benzene solution after 2 days at 281 K. Yield 50 mg (26%). ^1^H NMR (300.0 MHz, CDCl_3_): δ = 7.00 (m, 2 H, *meta*-Ph), 2.47 (br., 6 H, *ortho*-Me), 2.34 (br., 3 H, *para*-Me). ^13^C{1H} NMR (75.5 MHz, CDCl_3_): δ = 154.1 (*ipso*-Mes), 141.6 (*ortho*-Mes), 139.3 (*para*-Mes), 128.0 (*meta*-Mes), 25.8 (*ortho*-Me), 20.9 (*para*-Me). EI^+^ m/*z* (%): 396.1 (20.0) 397.1 (32.0) 398.1 (64.0) 399.1 (60.0) 400.1 (100.0) 401.1 (32.0) 402.1 (68.0) 403.1 (6.0) 404.1 (12.0) [*M*]^+^, calcd. for [*M*]^+^ 396.1 (30.8) 397.1 (54.4) 398.1 (100.0) 399.1 (93.1) 400.1 (95.5) 401.1 (45.8) 402.1 (25.5) 403.1 (11.0) 404.1 (20.9).

### Refinement

H atoms were refined using a riding model, with methyl C—H = 0.98 Å and aromatic C—H = 0.95 Å and with *U*_iso_(H) = 1.5*U*_eq_(C) for methyl-H or 1.2*U*_eq_(C) for aromatic-H. The methyl groups were allowed to rotate but not to tip. The methyl group in *para* position of the phenyl ring is disordered over two equally occupied positions.

### Crystal data

**Table:**

[HgBr(C_9_H_11_)]	*F*(000) = 720
*M**_r_* = 399.68	*D*_x_ = 2.649 Mg m^−^^3^
Monoclinic, *C*2/*c*	Mo *K*α radiation, λ = 0.71073 Å
Hall symbol: -C 2yc	Cell parameters from 8005 reflections
*a* = 10.0459 (8) Å	θ = 4.1–25.9°
*b* = 15.3072 (13) Å	µ = 19.28 mm^−^^1^
*c* = 8.1517 (7) Å	*T* = 173 K
β = 126.912 (5)°	Needle, colourless
*V* = 1002.27 (14) Å^3^	0.21 × 0.10 × 0.03 mm
*Z* = 4	

### Data collection

**Table:**

Stoe IPDS II two-circle diffractometer	942 independent reflections
Radiation source: Genix 3D IµS microfocus X-ray source	892 reflections with *I* > 2σ(*I*)
Genix 3D multilayer optics monochromator	*R*_int_ = 0.103
ω scans	θ_max_ = 25.6°, θ_min_ = 4.1°
Absorption correction: multi-scan (*MULABS*; Spek, 2009; Blessing, 1995)	*h* = −12→12
*T*_min_ = 0.107, *T*_max_ = 0.595	*k* = −18→18
6756 measured reflections	*l* = −9→9

### Refinement

**Table:**

Refinement on *F*^2^	Primary atom site location: structure-invariant direct methods
Least-squares matrix: full	Secondary atom site location: difference Fourier map
*R*[*F*^2^ > 2σ(*F*^2^)] = 0.025	Hydrogen site location: inferred from neighbouring sites
*wR*(*F*^2^) = 0.057	H-atom parameters constrained
*S* = 1.05	*w* = 1/[σ^2^(*F*_o_^2^) + (0.024*P*)^2^] where *P* = (*F*_o_^2^ + 2*F*_c_^2^)/3
942 reflections	(Δ/σ)_max_ = 0.001
55 parameters	Δρ_max_ = 1.19 e Å^−^^3^
0 restraints	Δρ_min_ = −0.71 e Å^−^^3^

### Special details

**Table:**

Experimental. ;
Geometry. All e.s.d.'s (except the e.s.d. in the dihedral angle between two l.s. planes) are estimated using the full covariance matrix. The cell e.s.d.'s are taken into account individually in the estimation of e.s.d.'s in distances, angles and torsion angles; correlations between e.s.d.'s in cell parameters are only used when they are defined by crystal symmetry. An approximate (isotropic) treatment of cell e.s.d.'s is used for estimating e.s.d.'s involving l.s. planes.
Refinement. Refinement of *F*^2^ against ALL reflections. The weighted *R*-factor *wR* and goodness of fit *S* are based on *F*^2^, conventional *R*-factors *R* are based on *F*, with *F* set to zero for negative *F*^2^. The threshold expression of *F*^2^ > σ(*F*^2^) is used only for calculating *R*-factors(gt) *etc*. and is not relevant to the choice of reflections for refinement. *R*-factors based on *F*^2^ are statistically about twice as large as those based on *F*, and *R*- factors based on ALL data will be even larger.

### Fractional atomic coordinates and isotropic or equivalent isotropic displacement parameters (Å^2^)

**Table:**

	*x*	*y*	*z*	*U*_iso_*/*U*_eq_	Occ. (<1)
Hg1	0.5000	0.600559 (17)	0.7500	0.03814 (13)	
Br1	0.5000	0.44177 (5)	0.7500	0.0531 (3)	
C1	0.5000	0.7347 (5)	0.7500	0.0336 (15)	
C2	0.6166 (6)	0.7798 (4)	0.7414 (7)	0.0331 (10)	
C3	0.6142 (7)	0.8712 (4)	0.7429 (8)	0.0375 (12)	
H3	0.6940	0.9024	0.7389	0.045*	
C4	0.5000	0.9178 (5)	0.7500	0.0369 (18)	
C5	0.7411 (7)	0.7320 (4)	0.7278 (9)	0.0422 (13)	
H5A	0.7982	0.6875	0.8359	0.063*	
H5B	0.8229	0.7736	0.7453	0.063*	
H5C	0.6831	0.7037	0.5933	0.063*	
C6	0.5000	1.0155 (5)	0.7500	0.048 (2)	
H6A	0.6116	1.0368	0.8599	0.073*	0.50
H6B	0.4202	1.0368	0.7726	0.073*	0.50
H6C	0.4682	1.0368	0.6175	0.073*	0.50

### Atomic displacement parameters (Å^2^)

**Table:**

	*U*^11^	*U*^22^	*U*^33^	*U*^12^	*U*^13^	*U*^23^
Hg1	0.03770 (18)	0.02853 (18)	0.0464 (2)	0.000	0.02430 (15)	0.000
Br1	0.0503 (5)	0.0291 (5)	0.0834 (7)	0.000	0.0419 (5)	0.000
C1	0.038 (4)	0.029 (3)	0.032 (4)	0.000	0.020 (3)	0.000
C2	0.030 (2)	0.038 (3)	0.026 (2)	−0.001 (2)	0.014 (2)	−0.003 (2)
C3	0.037 (3)	0.038 (3)	0.034 (3)	−0.007 (2)	0.019 (2)	−0.004 (2)
C4	0.043 (4)	0.021 (3)	0.032 (4)	0.000	0.014 (3)	0.000
C5	0.038 (3)	0.045 (3)	0.044 (3)	−0.003 (2)	0.025 (3)	−0.001 (2)
C6	0.060 (6)	0.033 (4)	0.045 (5)	0.000	0.028 (4)	0.000

### Geometric parameters (Å, º)

**Table:**

Hg1—C1	2.053 (7)	C4—C3^i^	1.381 (7)
Hg1—Br1	2.4307 (8)	C4—C6	1.495 (10)
C1—C2^i^	1.397 (6)	C5—H5A	0.9800
C1—C2	1.397 (6)	C5—H5B	0.9800
C2—C3	1.400 (8)	C5—H5C	0.9800
C2—C5	1.510 (7)	C6—H6A	0.9800
C3—C4	1.381 (7)	C6—H6B	0.9800
C3—H3	0.9500	C6—H6C	0.9800
			
C1—Hg1—Br1	180.000 (1)	C2—C5—H5A	109.5
C2^i^—C1—C2	120.8 (7)	C2—C5—H5B	109.5
C2^i^—C1—Hg1	119.6 (3)	H5A—C5—H5B	109.5
C2—C1—Hg1	119.6 (3)	C2—C5—H5C	109.5
C1—C2—C3	118.3 (5)	H5A—C5—H5C	109.5
C1—C2—C5	121.4 (5)	H5B—C5—H5C	109.5
C3—C2—C5	120.3 (5)	C4—C6—H6A	109.5
C4—C3—C2	122.4 (5)	C4—C6—H6B	109.5
C4—C3—H3	118.8	H6A—C6—H6B	109.5
C2—C3—H3	118.8	C4—C6—H6C	109.5
C3—C4—C3^i^	117.8 (7)	H6A—C6—H6C	109.5
C3—C4—C6	121.1 (3)	H6B—C6—H6C	109.5
C3^i^—C4—C6	121.1 (3)		
			
C2^i^—C1—C2—C3	0.4 (3)	C1—C2—C3—C4	−0.8 (7)
Hg1—C1—C2—C3	−179.6 (3)	C5—C2—C3—C4	178.4 (4)
C2^i^—C1—C2—C5	−178.8 (5)	C2—C3—C4—C3^i^	0.4 (4)
Hg1—C1—C2—C5	1.2 (5)	C2—C3—C4—C6	−179.6 (4)

Symmetry code: (i) −*x*+1, *y*, −*z*+3/2.
